# Distribution of human CYP2C8*2 allele in three different African populations

**DOI:** 10.1186/1475-2875-11-125

**Published:** 2012-04-25

**Authors:** Giacomo M Paganotti, Silvia Gramolelli, Francesca Tabacchi, Gianluca Russo, David Modiano, Mario Coluzzi, Rita Romano

**Affiliations:** 1 Department of Public Health and Infectious Diseases, Sapienza University, P.le Aldo Moro 5, 00185 Rome, Italy

**Keywords:** CYP2C8 enzyme, CYP2C8*2 allele, *Plasmodium falciparum *malaria, Africa, Poor metabolizers, Chloroquine, Amodiaquine, Drug-resistance

## Abstract

**Background:**

The aim of this study was to investigate cytochrome P450 2C8*2 (CYP2C8*2) distribution and allele frequency in three populations from West and East Africa exposed to *Plasmodium falciparum *malaria. CYP2C8 enzyme is involved in the metabolism of the anti-malarials amodiaquine and chloroquine. The presence of the CYP2C8*2 defective allele has been recently associated to higher rate of chloroquine-resistant malaria parasites.

**Methods:**

A total of 503 young subjects were genotyped for the single nucleotide polymorphism rs11572103 (A/T). Eighty-eight were from southern Senegal, 262 from eastern Uganda and 153 from southern Madagascar. The PCR-RFLP technique was used to discriminate the wild-type (A) from the defective allele (T).

**Results:**

A CYP2C8*2 (T) allele frequency of 0.222 ± 0.044 was detected in Senegal, 0.105 ± 0.019 in Uganda and 0.150 ± 0.029 in Madagascar.

**Conclusions:**

This study demonstrated that CYP2C8*2 allele is widespread in Africa. This allele occurs at different frequency in West and East Africa, being higher in Senegal than in Uganda and Madagascar. These data indicate that an important fraction of the populations analysed has a decreased enzymatic activity, thus being at higher risk for drug accumulation with two possible consequences: i) an exacerbation of drug-associated adverse side effects; ii) an increase of drug-resistance selection pressure on *P. falciparum *parasites.

## Background

*Plasmodium falciparum *malaria is one of the most important infectious diseases in the developing world, representing a priority in public health mainly in sub-Saharan Africa. Nowadays, anti-malarial strategies include: the development of a vaccine, the vector control, as well as drug treatment, which remains the most effective remedy to clear the infection. However, the spread of anti-malarial drug-resistance affects the outcome of treatments [1], since *P. falciparum *has selected resistant strains for the majority of the molecules used in anti-malarial therapy [2]. As recently demonstrated, host genetic variation in drug metabolizing enzymes influences the selection of *P. falciparum *drug-resistance in Burkina Faso [3]. In particular, the cytochrome P450 2 C8 (CYP2C8), a polymorphic enzyme that mainly contributes to the hepatic metabolism of amodiaquine (AQ) and chloroquine (CQ), shows a genetic variant (CYP2C8*2) that is associated with higher rate of drug-resistant parasites in the infected host (*pfcrt*-76Y and *pfmdr1*-86Y *P. falciparum *alleles) [3].

CYP2C8 is a member of the human CYP2C enzyme family, which also includes CYP2C9, CYP2C18 and CYP2C19, whose genes are located on the chromosome 10q24 [4]. Human CYP2C8 is involved in the metabolism of a variety of clinically important drugs, including the anti-malarials AQ, CQ and, to a lesser extent, dapsone (DDS) [4]. CYP2C8 is the only enzyme involved in the biotransformation of AQ [4], whereas for CQ it plays a major role [5,6], the secondary routes for CQ metabolism being limited by genetic and inhibitory factors in Africans [3,5-11]. *CYP2C8 *gene is known to be polymorphic, and the distribution of variant alleles differs among ethnic populations [4]. CYP2C8*2, the variant most common in Africans, is related to a poor metabolizer phenotype (PM) in subjects carrying at least one copy of the defective allele [4,9]. Subjects who are poor metabolizers experience a longer drug half-life [12] and have increased adverse side effects. In particular, CYP2C8*2 shows six fold lower intrinsic clearance of AQ than wild type [12]. On the other hand, no evidence is yet available from the literature about the role of *CYP2C8 *genetic variance in CQ pharmacokinetics [13], although there is indirect evidence of lower CQ metabolism in CYP2C8*2-carriers shown through the association between the allele and rates of CQ-resistant *P. falciparum *parasites [3]. In humans, CQ concentrations decline multi-exponentially and elimination at its initial anti-malarial concentrations is relatively rapid. This means that it usually persists for only a few days at concentrations sufficient to select resistant over sensitive parasites (its window of selection). The poor metabolizers obviously greatly extent these windows. A slower metabolism of an anti-malarial drug leads to a longer time of parasite exposure to a sub-therapeutic level of the molecule, therefore acting as a further co-factor in drug-resistance selection. Few studies had described the CYP2C8*2 allele frequency in Africa, and the actual knowledge of its distribution is incomplete all over the continent. The prevalence of this allele is reported to be 13.9% in Zanzibar [5], whereas in Ghana it ranges between 16.8% and 17.9% [14-16]. Two studies had been conducted in Burkina Faso, the former showed a CYP2C8*2 prevalence of 11.5% in the south of the country [12]. In the second study, sympatric ethnic groups living in the central area of Burkina Faso were analysed: the Fulani showed a prevalence of 9.9% and the Mossi-Rimaibè group 23.7% [3]. Moreover, Dai et al. [17] reported a value of 18% in African-Americans. CYP2C8*2 is virtually absent in non-African populations [17], as in Caucasians, where instead the poor metabolizer allele is represented by CYP2C8*3, which is absent or found at very low frequency in Africa [17].

Actually, the official policies for the treatment of uncomplicated forms of *P. falciparum *malaria are based on artemisinin combination therapy (ACT), including the association of artesunate with AQ. However, CQ is still used as anti-malarials in several African countries [18-22]. Moreover, the CQ tablets are often of poor quality [23,24] and the compliance to the therapy is low and then the selective effects on parasite are improved.

The aim of the study was to describe the distribution of CYP2C8*2 in Senegal, Uganda and Madagascar, areas that were not investigated by previous studies and characterized by different levels of *P. falciparum *malaria transmission intensity.

## Methods

### Study area and subjects

**Figure 1:**
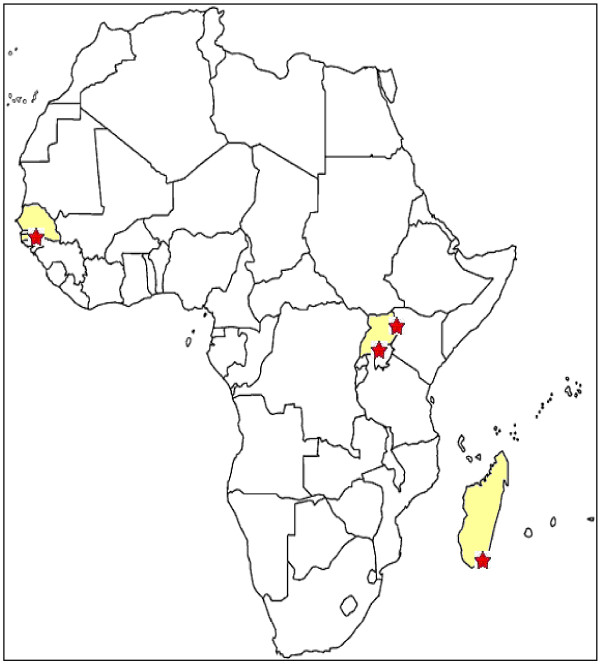
**Study areas**.

The samples analysed in the present study were collected during cross-sectional surveys performed during 2007 in Senegal (Thionck Essyl health centre - Casamance region); Uganda (Namalu and Rupa health centres, Kakoliye and Nadunget primary schools - Karamoja region; Makindiye children centre - Kampala region); and Madagascar (Amboasary, Antsanomaro, Masihanaka and Fort Dauphin health centres - Anosy region) (Figure 1). A total of 503 children and adolescents had been enrolled: 88 were from Senegal (mean age in years ± SD = 10.4 ± 3.5); 262 from Uganda (mean age 7.8 ± 3.0) and 153 from Madagascar (mean age 6.0 ± 4.1). For the purpose of the study were considered only unrelated individuals according to different family names and interviews. The same protocol for enrollment was followed in all sites [25,26]. Signed informed consent for multiple genetic and epidemiologic surveys was obtained from the subjects or their parents/caregivers [25,26]. This study was conducted with the approval of the ethics committee and research committee of the Sapienza University of Rome. Fingerpick blood samples were spotted on Whatman grade 1 filter papers at the time of the field survey and then air dried before being separately stored in sealed plastic containers.

### Genotyping

Human DNA was extracted with Chelex-100 resin (Bio-Rad). CYP2C8*2 (rs11572103, A > T) detection was carried out using the PCR-RFLP technique. Two μl of DNA template were used to amplify by PCR a 107 bp fragment of the *CYP2C8 *gene (forward primer: 5'-GAACACCAAGCATCACTGGA-3'; reverse primer: 5'-GAAATCAAAATACTGATCTGTTGC-3'); the PCR product was then incubated with *Bcl*I enzyme that cuts the wild-type allele only (A); undigested products represent the variant allele (T). To detect the size polymorphisms, the samples were ran on a Metaphor 3% gel. Controls for human genotyping were utilized after sequencing of the PCR product obtained from each different genotype. Moreover, to avoid genotyping error, the analysis was repeated for all the heterozygous samples.

### Statistical analysis

Inter-populations comparisons were obtained by Yates-corrected *χ*^2 ^test and by Mantel-Haenszel *χ*^2 ^test (M-H). Odds ratios (ORs) were calculated with 95% confidence intervals (CIs). The analyses were performed with epi info 6 statistical package. GenePop software was used for the evaluation of hardy-Weinberg equilibrium [27].

## Results

The frequencies of the CYP2C8*2 allele for each country included in the present study are reported in Table 1. Genotype frequencies were in Hardy-Weinberg equilibrium both for Uganda and Madagascar (*χ*^2 ^= 1.52, *P *= 0.221 and *χ*^2 ^= 2.61, *P *= 0.106, respectively), while an excess of AT heterozygotes was found for Senegal (*χ*^2 ^= 7.11, *P *= 0.008), probably because of the small sample size (N = 88). Comparing the allele frequency among the populations studied, a statistically significant difference was found between Senegal and Uganda (OR = 2.43, 95% CI 1.51-3.91; Yates-corrected *χ*^2 ^= 14.43, *P *= 0.0001; M-H *χ*^2 ^= 15.49, *P *< 0.0001) whereas a lower difference was revealed in the other comparisons (Senegal *vs *Madagascar: OR = 1.85, 95% CI 1.11-3.10; Yates-corrected *χ*^2 ^= 5.62, *P *= 0.0177; M-H *χ*^2 ^= 6.23, *P *= 0.0126; Madagascar *vs *Uganda: OR = 0.76, 95% CI 0.48-1.20; Yates-corrected *χ*^2 ^= 1.24, *P *= 0.2654; M-H *χ*^2 ^= 1.50, *P *= 0.2201). Furthermore, the percentage of subjects carrying at least one copy of the CYP2C8*2 allele (T) was 44.3%, 20.6% and 26.1% for Senegal, Uganda and Madagascar, respectively.

**Table 1:** *CYP2C8 *genotypes and T allele frequency in three African populations

Countries		*CYP2C8 *(rs11572103, A > T) frequencies			
		
		Relative and (absolute) genotype frequencies			Allele frequency ± SE
**Sample**	**N**	**AA**	**AT**	**TT**	**T**

Senegal	88	0.557 (49)	0.443 (39)	0.000 (0)	0.222 ± 0.044

Uganda	262	0.797 (208)	0.199 (53)	0.004 (1)	0.105 ± 0.019

Madagascar	153	0.739 (113)	0.222 (34)	0.039 (6)	0.150 ± 0.029

## Discussion

The discovery of functional variability in genes encoding drug metabolizing enzymes has contributed significantly to the understanding of the inter-individual variability in dose-concentration relationships and drug response. Knowledge of polymorphisms in genes encoding enzymes that metabolize anti-malarial drugs, as well as their associations with clinical and parasitological outcomes, can be useful in designing dosage regimens and modulating drug therapy that is safe, effective and therefore less likely to select for pathogen's drug resistance.

This work describes the distribution of the African defective allele CYP2C8*2, whose corresponding enzyme is an important player in the metabolism of two widespread anti-malarial compounds, CQ and AQ. The allele frequencies of CYP2C8*2 observed in the three different populations are partially in agreement with other data available in the literature. In particular, the allele frequency found in Uganda (10.5%) is in line with that reported from Zanzibar (13.9%), to date the only other study conducted in East Africa [9]. In Uganda, the Karamoja region and the suburban districts of the capital Kampala are areas of hyper-endemic malaria transmission [28], where anti-malarial treatment is based on ACT, mainly a combination of artemether and lumefantrine (AL) and, as an alternative, artesunate (AS) plus AQ [28]. Consequently, the presence of the CYP2C8*2 allele may be a potential co-factor in the onset of adverse side effects associated with AQ administration. Furthermore, as there is evidence that both *pfcrt *and *pfmdr1 *parasite resistant alleles play a role in AQ-resistance [29], it could emerge the risk related to the presence of CYP2C8*2 in selecting AQ- resistant strains, since the interplay between host and parasite genetic variation could be similar to that of CQ.

No data of *CYP2C8 *polymorphisms in Madagascar are available in the literature. Here it is reported a non-negligible frequency of 26.1% of CYP2C8*2-carriers in an area of low malaria transmission, where CQ was still used for therapy and prophylaxis although it has been replaced by AS plus AQ combination therapy as the first-line treatment for uncomplicated cases since 2005 [30]. In Madagascar, *P. falciparum *CQ-resistance is mainly based on *pfmdr1*-86Y rather than *pfcrt*-76Y alleles [30,31]. Intriguingly, this setting is similar to that previously reported from Burkina Faso, where the observation of CQ-resistance was mainly based on *pfmdr1 *polymorphism [3].

Concerning Senegal, the excess of AT heterozygotes could be attributable to a stochastic effect due to the sampling, despite the efforts to avoid the enrolment of related subjects. Nevertheless, a deviation of the genotype frequencies from the expected Hardy-Weinberg proportions could be expected in discrete populations [32]. The CYP2C8*2 allele frequency found in the Senegalese subjects (22.2%) is similar to that of Mossi-Rimaibè from Burkina Faso (23.7%) [3] and, in general, the frequency of this allele is higher in West than in East Africa, two-fold higher in Senegal than in Uganda, as reported in this study.

## Conclusions

Although malaria infection in Africa is associated with a perverse cycle of disease and poverty that hampers most eradication and control efforts, the study of pharmacogenetics of anti-malarial drugs in human populations exposed to *P. falciparum *suggests that an early identification of PM individuals could lead to alternative therapy of selected individuals/groups in order to minimize the adverse side effects (and therefore the compliance to therapy) as well as delay the spread of drug-resistance.

## Competing interests

The authors declare that they have no competing interests.

## Authors' contributions

GMP and RR conceived the study. GMP, FT, DM, MC and RR coordinated the study. GMP, SG and FT carried out the molecular genetic study, performed database and statistical analyses. GR carried out field surveys. GMP, SG, FT and RR drafted the manuscript. All authors read and approved the final manuscript.

## References

[B1] Hastings IM Korenromp EL Bloland PB The anatomy of a malaria disaster: drug policy choice and mortality in African children Lancet Infect Dis 2007 7 739 748 10.1016/S1473-3099(07)70214-1 17884732

[B2] Mita T Tanabe K Kita K Spread and evolution of *Plasmodium falciparum *drug resistance Parasitol Int 2009 58 201 209 10.1016/j.parint.2009.04.004 19393762

[B3] Paganotti GM Gallo BC Verra F Sirima BS Nebié I Diarra A Coluzzi M Modiano D Human genetic variation is associated with *Plasmodium falciparum *drug-resistance J Infect Dis 2011 204 1772 1778 10.1093/infdis/jir629 21998472

[B4] Totah RA Rettie AE Cytochrome P450 2 C8: substrates, inhibitors, pharmacogenetics, and clinical relevance Clin Pharmacol Ther 2005 77 341 352 10.1016/j.clpt.2004.12.267 15900280

[B5] Kim KA Park JY Lee JS Lim S Cytochrome P450 2 C8 and CYP3A4/5 are involved in chloroquine metabolism in human liver microsomes Arch Pharm Res 2003 26 631 637 10.1007/BF02976712 12967198

[B6] Projean D Baune B Farinotti R Flinois JP Beaune P Taburet AM Ducharme J In vitro metabolism of chloroquine: identification of CYP2C8, CYP3A4, and CYP2D6 as the main isoforms catalyzing N-desethylchloroquine formation Drug Metab Dispos 2003 31 748 754 10.1124/dmd.31.6.748 12756207

[B7] Shimada T Yamazaki H Mimura M Inui Y Guengerich FP Interindividual variations in human liver cytochrome P-450 enzymes involved in the oxidation of drugs, carcinogens and toxic chemicals: studies with liver microsomes of 30 Japanese and 30 Caucasians J Pharmacol Exp Ther 1994 270 414 423 8035341

[B8] Zeigler-Johnson CM Walker AH Mancke B Spangler E Jalloh M McBride S Deitz A Malkowicz SB Ofori-Adjei D Gueye SM Rebbeck TR Ethnic differences in the frequency of prostate cancer susceptibility alleles at SRD5A2 and CYP3A4 Hum Hered 2002 54 13 21 10.1159/000066695 12446983

[B9] Cavaco I Strömberg-Nörklit J Kaneko A Msellem MI Dahoma M Ribeiro VL Bjorkman A Gil JP CYP2C8 polymorphism frequencies among malaria patients in Zanzibar Eur J Clin Pharmacol 2005 61 15 18 10.1007/s00228-004-0871-8 15785959

[B10] Garsa AA McLeod HL Marsh S CYP3A4 and CYP3A5 genotyping by Pyrosequencing BMC Med Genet 2005 9 6 19 10.1186/1471-2350-6-19 PMC1142317 15882469

[B11] Penzak SR Kabuye G Mugyenyi P Mbamanya F Natarajan V Alfaro RM Kityo C Formentini E Masur H Cytochrome P450 2B6 (CYP2B6) G516T influences nevirapine plasma concentrations in HIV-infected patients in Uganda HIV Med 2007 8 86 91 10.1111/j.1468-1293.2007.00432.x 17352764

[B12] Parikh S Ouedraogo JB Goldstein JA Rosenthal PJ Kroetz DL Amodiaquine metabolism is impaired by common polymorphisms in CYP2C8: implications for malaria treatment in Africa Clin Pharmacol Ther 2007 82 197 203 10.1038/sj.clpt.6100122 17361129

[B13] Mehlotra RK Henry-Halldin CN Zimmerman PA Application of pharmacogenomics to malaria: a holistic approach for successful chemotherapy Pharmacogenomics 2009 10 435 449 10.2217/14622416.10.3.435 19290792 PMC2717014

[B14] Röwer S Bienzle U Weise A Lambertz U Forst T Otchwemah RN Pfützner A Mockenhaupt FP Short communication: high prevalence of the cytochrome P450 2 C8*2 mutation in Northern Ghana Trop Med Int Health 2005 10 1271 1273 10.1111/j.1365-3156.2005.01525.x 16359408

[B15] Adjei GO Kristensen K Goka BQ Hoegberg LC Alifrangis M Rodrigues OP Kurtzhals JA Effect of concomitant artesunate administration and cytochrome P4502C8 polymorphisms on the pharmacokinetics of amodiaquine in Ghanaian children with uncomplicated malaria Antimicrob Agents Chemother 2008 52 4400 4406 10.1128/AAC.00673-07 18779360 PMC2592852

[B16] Kudzi W Dodoo AN Mills JJ Characterisation of CYP2C8, CYP2C9 and CYP2C19 polymorphisms in a Ghanaian population BMC Med Genet 2009 10 124 129 10.1186/1471-2350-10-124 19954515 PMC3224726

[B17] Dai D Zeldin DC Blaisdell JA Chanas B Coulter SJ Ghanayem BI Goldstein JA Polymorphisms in human CYP2C8 decrease metabolism of the anticancer drug paclitaxel and arachidonic acid Pharmacogenetics 2001 11 597 607 10.1097/00008571-200110000-00006 11668219

[B18] Frosch AE Venkatesan M Laufer MK Patterns of chloroquine use and resistance in sub-Saharan Africa: a systematic review of household survey and molecular data Malar J 2011 10 116 10.1186/1475-2875-10-116 21554692 PMC3112453

[B19] Sarrassat S Lalou R Cissé M Le Hesran JY Management of uncomplicated malaria in children under 13 years of age at a district hospital in Senegal: from official guidelines to usual practices Malar J 2011 10 285 10.1186/1475-2875-10-285 21958422 PMC3204280

[B20] O'Connell KA Gatakaa H Poyer S Njogu J Evance I Munroe E Solomon T Goodman C Hanson K Zinsou C Akulayi L Raharinjatovo J Arogundade E Buyungo P Mpasela F Adjibabi CB Agbango JA Ramarosandratana BF Coker B Rubahika D Hamainza B Chapman S Shewchuk T Chavasse D Got ACTs? Availability, price, market share and provider knowledge of anti-malarial medicines in public and private sector outlets in six malaria-endemic countries Malar J 2011 10 326 10.1186/1475-2875-10-326 22039838 PMC3227612

[B21] Littrell M Gatakaa H Evance I Poyer S Njogu J Solomon T Munroe E Chapman S Goodman C Hanson K Zinsou C Akulayi L Raharinjatovo J Arogundade E Buyungo P Mpasela F Adjibabi CB Agbango JA Ramarosandratana BF Coker B Rubahika D Hamainza B Shewchuk T Chavasse D O'Connell KA Monitoring fever treatment behaviour and equitable access to effective medicines in the context of initiatives to improve ACT access: baseline results and implications for programming in six African countries Malar J 2011 10 327 10.1186/1475-2875-10-327 22039892 PMC3223147

[B22] World Health Organization World malaria report. Geneva 2011

[B23] Sawadogo CW Amood Al-Kamarany M Al-Mekhlafi HM Elkarbane M Al-Adhroey AH Cherrah Y Bouklouze A Quality of chloroquine tablets available in Africa Ann Trop Med Parassito 2011 105 10.1179/1364859411Y.0000000030 PMC4100301 22117854

[B24] Newton PN Green MD Mildenhall DC Plancon A Nettey H Nyadong L Hostetler DM Swamidoss I Harris GA Powell K Timmermans AE Amin AA Opuni SK Barbereau S Faurant C Soong RC Faure K Thevanayagam J Fernandes P Kaur H Angus B Stepniewska K Guerin PJ Fernandez FM Poor quality vital anti-malarials in Africa - an urgent neglected public health priority Malar J 2011 10 352 10.1186/1475-2875-10-352 22152094 PMC3262771

[B25] Romano R Tabacchi F Paganotti GM Russo G Gramolelli S Marinucci F Ceccherini-Nelli L Coluzzi M Evaluation of bloodsucking arthropod bite as possible risk co-factor in *Human herpesvirus*-8 transmission route Parassitologia 2010 52 417 422 22320016

[B26] Romano R Gramolelli S Tabacchi F Russo G Verzaro S Marinucci F Paganotti GM Gaeta A Coluzzi M Human Herpesvirus 8 (HHV-8): salivary shedding in mothers and children from Uganda: risk factors and clues about transmission Prevention and Research 2011 1 44 52

[B27] http://genepop.curtin.edu.au/

[B28] Yeka A Gasasira A Mpimbaza A Achan J Nankabirwa J Nsobya S Staedke SG Donnelly MJ Wabwire-Mangen F Talisuna A Dorsey G Kamya MR Rosenthal PJ Malaria in Uganda: challenges to control on the long road to elimination: I. Epidemiology and current control efforts Acta Trop 2012 121 184 195 21420377 10.1016/j.actatropica.2011.03.004 PMC3156969

[B29] Echeverry DF Holmgren G Murillo C Higuita JC Björkman A Gil JP Osorio L Short report: polymorphisms in the pfcrt and pfmdr1 genes of *Plasmodium falciparum *and in vitro susceptibility to amodiaquine and desethylamodiaquine Am J Trop Med Hyg 2007 77 1034 1038 18165517

[B30] Andriantsoanirina V Ratsimbasoa A Bouchier C Tichit M Jahevitra M Rabearimanana S Raherinjafy R Mercereau-Puijalon O Durand R Ménard D Chloroquine clinical failures in *P. falciparum *malaria are associated with mutant Pfmdr-1, not Pfcrt in Madagascar PLoS One 2010 5 e13281 10.1371/journal.pone.0013281 20967251 PMC2954150

[B31] Rason MA Andrianantenaina HB Ariey F Raveloson A Domarle O Randrianarivelojosia M Prevalent pfmdr1 n86y mutant *Plasmodium falciparum *in Madagascar despite absence of pfcrt mutant strains Am J Trop Med Hyg 2007 76 1079 1083 17556614

[B32] Wang J Estimation of effective population sizes from data on genetic markers Phil Trans R Soc B 2005 360 1395 1409 10.1098/rstb.2005.1682 16048783 PMC1847562

